# From Perceived Supervisor Social Power to Employee Commitment: Definition and Scale Development

**DOI:** 10.3389/fpsyg.2021.603739

**Published:** 2021-02-26

**Authors:** Léandre Alexis Chénard-Poirier, Christian Vandenberghe, Alexandre J. S. Morin

**Affiliations:** ^1^Research Laboratory on Social Behavior, Département de Psychologie, Université du Québec à Montréal, Montreal, QC, Canada; ^2^HEC Montréal, Montreal, QC, Canada; ^3^Substantive-Methodological Synergy Research Laboratory, Department of Psychology, Concordia University, Montreal, QC, Canada

**Keywords:** perceived supervisor social power, organizational commitment affective, commitment to supervisors, power bases, turnover intention, industrial and organizational psychology

## Abstract

It has been theoretically proposed that employees’ perceptions of their supervisor social power in the organization entail a potential to influence their beliefs, attitudes, and behaviors. However, no study has investigated such potential. This lack of research stems from the absence of a common understanding around the meaning of perceived supervisor social power (PSSP) and the absence of any validated measure. Therefore, the purpose of this article is to establish PSSP definition and to validate a five-item scale to measure this construct. Three studies encompassing four independent samples of employees from three different countries and three different languages (i.e., France, cross-sectional [*Study 1*, Sample 1], Canada, cross-sectional [*Study 1*, Sample 2: French Canada; *Study 2*: English Canada], Romania, two-wave data collection [*Study 3*]) were conducted to assess the validity of PSSP. Results showed that responses to the PSSP scale presented excellent psychometric properties (i.e., factor validity, reliability, and convergent and discriminant validity). Furthermore, the structure of the proposed five-item measure of PSSP was found to be invariant across four samples. Finally, PSSP nomological validity (i.e., integration into a nomological network) was assessed. *Study* 1 and *Study 2* showed that PSSP was positively related to affective organizational commitment. All three studies showed that PSSP acted as a positive moderator of the relation between affective commitment to the supervisor and affective organizational commitment. Together, these studies support the psychometric soundness of the PSSP scale and presented the first evidence of its potential to influence followers. Implications of these findings for future research on supervisor social power are discussed.

## Introduction

Social power is generally defined as the potential to influence another person (French and Raven, 1959; Raven, 1992, 2008). This potential entails that a person may change his/her beliefs, attitudes or behaviors as a result of the actions, or simply the presence, of an influencing agent (Kelman, 1958, 1961; Raven, 1992, 2008). Power has long been considered a foundational concept to the understanding of social relations and as a central component of many human interactions (Russell, 1938; Mittal and Elias, 2016). Power is an even more important concept in management as it is used to influence followers in order to achieve organizational goals and lies at the core of the supervisor’s role (Yukl et al., 1993; Northouse, 2013). Consequently, a wealth of research has investigated social power in the context of the supervisor-follower dyad (Yukl and Falbe, 1991; Yukl and Tracey, 1992; Carson et al., 1993; Yukl et al., 1993; Elangovan and Xie, 1999; Koslowsky et al., 2001; Pierro et al., 2013; Chi and Ho, 2014; Chiu et al., 2017; Chi et al., 2018; Haller et al., 2018; Peyton et al., 2019). This research, however, has primarily focused on French and Raven’s (1959) power bases (i.e., reward, coercive, legitimate, expert, and referent), which refer to types of tactics used by supervisors to directly influence their followers (Raven, 1992, 2008). For example, studies showed that when power bases are considered together as a global perception of the supervisor’s social power, they are positively related to perceived leadership effectiveness (Chiu et al., 2017) and to followers’ organizational commitment, performance, job satisfaction, and work engagement (Haller et al., 2018). When considered individually, research has shown that the positive relations between social power bases and follower outcomes were mostly present when supervisors used power bases that did not heavily rely on compliance (e.g., coercive), but let followers the freedom to accept influence attempts (e.g., expert or reference; Pierro et al., 2013; Peyton et al., 2019).

Albeit informative, these studies have mainly considered the effects of social power on followers’ beliefs, attitudes, and behaviors as a result of direct and intentional attempts to influence them. This literature thus ignores that followers also develop a general perception of their supervisor’s social power that integrates all available information, including not only influence attempts directed toward them but also by observing influence attempts directed at others (Fiol et al., 2001). It also ignores the possibility that these indirect observations might also have an influence on subordinates. The lack of research on these indirect perceptions of supervisor’s social power limits our understanding of how followers construct their global perception of their supervisor’s power.

Farmer and Aguinis (2005) suggested that followers’ perceptions of their manager’s ability to influence important organizational actors or the organization as a whole might be of particular importance when forming perceptions pertaining to their supervisor’s social power. Thus, although located outside of the supervisor-follower relationship, employees’ perceptions of their supervisor’s social power (PSSP) could have the potential to influence followers’ beliefs, attitudes, and behaviors. Specifically, through identification and internalization mechanisms, association with a powerful supervisor might progressively lead followers to view themselves as more powerful agents in the organization (Fiol et al., 2001), in turn bringing them to embrace organizational goals and values as their own. To our knowledge, despite some theoretical propositions in this area, no study has looked at PSSP potential to influence followers. This gap in research stems both from the absence of a clear definition of PSSP, and from the absence of any validated measure of PSSP.

In the following sections we first define the concept of PSSP. We then conceptually distinguish PSSP from related constructs (i.e., power bases, perceived supervisor networking ability, employees’ perceptions of supervisor organizational support, and perceived supervisor-organization value congruence) and propose a nomological network involving affective commitment to the organization and the supervisor, and turnover intention. Then, following MacKenzie et al. (2011) recommended stages of scale development, we developed a short measure of PSSP and formally specify its measurement model. We then assessed the factor structure, reliability, convergent and discriminant validity, and the nomological validity of this new PSSP scale. These empirical assessments were cross-validated in three studies encompassing four independent samples of employees from three different countries and three different languages (i.e., France [*Study 1*, Sample 1], Canada [*Study 1*, Sample 2: French Canada; *Study 2*: English Canada], Romania [*Study 3*]). Finally, we discuss the theoretical implications of the PSSP scale for research on power.

## Perceived Supervisor Social Power: Conceptual Definition and Influence Mechanism

### Defining Perceived Supervisor Social Power

Perceived supervisor social power is defined as *the global perception by a follower of his/her supervisor potential to influence important organizational actors and the organizational decision-making process*. First, this definition is consistent with and stems from the definition of social power (French and Raven, 1959; Raven, 1992, 2008) positioning PSSP as a perceived potential to influence others in the organization.

However, this definition entails that the direct receivers of this influence are not the followers themselves, but rather other major parties in the organization. Thus, PSSP reflects a third-party observation made by a follower. In addition, followers might not be privy to the specific influence tactics or power bases used by their supervisor to achieve and maintain this social power. However, employees have the ability to develop generic perceptions of their supervisor’s power, influence and status in the organization (Fiol et al., 2001; Eisenberger et al., 2002; Farmer and Aguinis, 2005) irrespective of their own dyadic relationship with their supervisor. This definition thus positions PSSP as a source of social information that employees can derive from their supervisors’ use of power directed at other important organizational actors, and that allows them to appraise their supervisor’s centrality in the organizational decision-making process (Eisenberger et al., 2002; Venkataramani et al., 2010; Chi et al., 2018). For example, an employee may not know how his/her supervisor behaves in an important meeting regarding the company strategy but may know that his/her vision has been adopted by the organization.

Therefore, PSSP is conceptualized as a unidimensional construct pertaining to a follower’s global perception of his/her supervisor social power in the organization. This conceptual definition served as the basis for the items’ generation process (MacKenzie et al., 2011). In the next section, we propose that PSSP entails a potential to influence employees’ attitudes, beliefs and behaviors, thus clarifying how it contributes to the understanding of influence dynamics in organizational settings.

### PSSP Influence Mechanism

Supervisors can influence employees through compliance, identification, and internalization (Kelman, 1958, 1961). Through compliance, followers do what their supervisor wants in order to obtain rewards or to avoid punishments. In contrast, as it is located outside of the supervisor-follower dyad, PSSP does not involve any request, reward, or punishment made by a supervisor to a subordinate. In fact, it does not even involve attempts to influence a subordinate. Therefore, PSSP should not involve compliance. PSSP’s potential influence on followers should thus emerge from identification and internalization mechanisms, stemming from social information, that do not require an actual interpersonal relationship or interactions between the employee and the person s/he identifies with (Ashforth et al., 2016).

Identification refers to attempts “to be like or actually be the other person” (Kelman, 1961, p. 63). First, powerful supervisors are attractive individuals who invite attention and are often seen as possessing characteristics that are desired by followers. From the follower’s point of view, PSSP could thus be considered as an agentic characteristic. Such attributions are related to individual characteristics like ambition, goal orientation or independence (Abele and Wojciszke, 2007; Gartzia and Baniandrés, 2016). It has been shown that in relationships where one person’s well-being and goal pursuit are dependent on the other person, such as in a supervisor-follower relationship, the dependent person will pay close attention to the agentic characteristics of the other and judge them as important (Abele and Wojciszke, 2007).

Second, through identification, followers may consciously or unconsciously develop more positive perceptions of themselves by internalizing the desired attribute of another person (Ashforth et al., 2016), a process also referred to as self-expansion (Aron et al., 2001). Identification may occur via a compensatory process where the integration of an attractive attribute (e.g., power) seeks to fulfill a need for self-enhancement. Through identification to powerful individuals or groups, followers may come to consider themselves as more powerful, and thus as more influential agents in their organization (Fiol et al., 2001). For example, a follower contributing to an organization-wide project may come to believe that his or her voice should have more weight due to the notoriety of his or her supervisor. Similarly, s/he may believe that the work of his or her team will be more visible and impactful in the organization as his or her supervisor is perceived as a central figure in the organization.

Moreover, employees are also more likely to internalize attributes that are perceived to be viable, desirable, and useful in a specific work context (Ashforth et al., 2016). As powerful supervisors are more salient representatives of the organization in the eyes of followers (Eisenberger et al., 2002; Venkataramani et al., 2010), employees should be more likely to internalize the organizational values and goals that they embody. Seeing themselves as more powerful and sharing the organization’s goals and values, employees are more likely to adopt positive beliefs, attitudes and behaviors toward their organization.

## Convergent and Discriminant Validity of the Perceived Supervisor Social Power

In the process of validating a new scale, it is important to assess whether the new measure is related, yet distinct, from measures of constructs that share the same conceptual space (MacKenzie et al., 2011). We first start by presenting how PSSP shares conceptual space with, and yet remains distinct from, power bases as they both entail a potential of influence (indirect for the former and direct for the latter). Then, as PSSP implies that the supervisor can have an influence over the organization, we also assessed PSSP’s convergence and distinctiveness with other constructs involving a relationship between the supervisor and the global organization. For this purpose, we considered the supervisor’s networking ability, the supervisor’s perceived organizational support, and perceptions of supervisor-organization value congruence. These relations were examined to test PSSP’s convergent and discriminant validity.

### PSSP and Power Bases

Perceived supervisor social power is conceptually distinct from, albeit related to, power bases. French and Raven’s (1959) power bases (i.e., coercive, reward, expert power, legitimate, and referent) provide a framework that helps delineate a supervisor’s ability to change the attitudes and behaviors of another person (or the ability to “enforce one’s will;” Sturm and Antonakis, 2015). According to Raven (1992), power bases involve a sense of volition at both ends of the influence process. Specifically, it is suggested that the supervisor is a rational agent that has a motivation to influence a follower. Thus, s/he deliberately selects the most appropriate power base(s) to influence a follower based on the availability of those bases and a cost-benefit analysis. Subsequently, followers are not viewed as passive targets of influence as they deliberately decide whether they will accept, or resist, attempts made to influence them. In contrast, PSSP does not involve a supervisor’s volition or action, but rather his or her ability to influence organizational functioning as perceived by employees. Likewise, PSSP does not involve any direct influence (volitional or not) of the supervisor on followers. Finally, the identification mechanism underlying PSSP’s potential to influence does not necessarily involve a conscious decision by a follower to be influenced by the supervisor (Ashforth et al., 2016). In short, power bases and PSSP both involve a potential to influence. However, power bases rely on direct influence attempts from the supervisor to the employee, while PSSP’s influence potential is a by-product of employees’ perceptions of the supervisor’s capacity to influence important organizational actors.

Perceived supervisor social power is also qualitatively related, yet distinct, from each specific power base when considered from the perspective of the target follower. *Coercive power* refers to the ability to administer punishments, while *reward power* reflects the ability to provide incentives to subordinates (Hinkin and Schriesheim, 1989, 1994). PSSP does not involve attempts to reward or punish the target employee. Likewise, *expert power* is based on the follower’s perception that the supervisor has specific knowledge and expertise related to his or her job that can be shared (Hinkin and Schriesheim, 1989, 1994; Atwater and Yammarino, 1996). Again, PSSP does not directly imply that a supervisor has a valued expertise or preferential information as s/he might have acquired his or her power in the organization through other means. However, employees might be more attentive to a powerful supervisor, and be more receptive to reward, coercive, and expert power used by such a supervisor.

*Legitimate power* emanates from the subordinate’s acceptance of the right of the supervisor to influence him or her and as such implies that the subordinate recognizes his/her obligation to comply (Hinkin and Schriesheim, 1989, 1994). Holding a formal structural position in the organization does mean that a supervisor has the right to ask followers to comply with their instructions. However, it does not imply that he has influence over people occupying a similar position or rank, or even located at upper levels of the hierarchy. For example, a supervisor from an isolated regional branch of a retail chain might have little influence over the organization decision process, thus low PSSP. However, his/her followers would acknowledge that s/he holds legitimate power from its supervisory position. Finally, according to Raven (1992), *referent power* stems from identification with an influencing agent. In the context of a supervisor-employee dyad, Hinkin and Schriesheim (1989, 1994) posited that it reflects the ability to obtain personal acceptance or approval from others (Hinkin and Schriesheim, 1989, 1994). Furthermore, Yukl et al. (1996) have shown that subordinates of supervisors relying on referent power might comply with their requests because of a friendship and out of a desire to maintain the relationship. PSSP and referent power should thus be related as they both entail identification to the supervisor. However, PSSP does not involve behaviors aimed at developing a sense of acceptance and approval by the follower.

In sum, although PSSP might emerge from supervisors’ ability to rely on various power bases to influence the larger organizational system, it remains distinct from these power bases:

*Hypothesis 1:* PSSP will be positively related to, yet distinct from, French and Raven’s (1959) bases of (a) reward, (b) coercive, (c) expert, (d) legitimate, and (e) referent power.

### PSSP and Supervisor-Organization Relations Constructs

As PSSP evokes the influence of the supervisor within the organization’s network, it is tied to the relationship between the supervisor and the organization. This relationship has attracted scholars’ attention for years. First, some studies have addressed this relationship through the degree of overlap in identities. Eisenberger et al. (2002) found that when supervisors are perceived to hold a high organizational status, the relation between employees’ perception of the support they receive from their supervisor and the organization was stronger. Likewise, Vandenberghe et al. (2017) reported that high supervisor-organization value congruence was associated with a stronger relation between affective commitment to the supervisor and organizational commitment. Second, studies have looked at the supervisor-organization relationship through the lens of the organization’s attitudes toward supervisors. For example, Erdogan and Enders (2007) found that strong perceptions of organizational support directed at the supervisor were associated with stronger relations between leader-member exchange and followers’ job satisfaction and performance. Similarly, Shanock and Eisenberger (2006) reported that subordinates’ perceptions of supervisor support increased their perceptions of organizational support and performance when perceptions of organizational support directed at the supervisor were stronger.

Although the above studies highlight important aspects of the supervisor-organization relationship, they do not delve into the proactive skills that would facilitate the emergence of PSSP. Indeed, research assumes that social power emerges from proactive attempts to influence others (Raven, 1992; Nesler et al., 1999). Political skills such as networking ability (i.e., a proactive ability to build a strong network and alliances, and to take advantage of opportunities; Ferris et al., 2005) provide a basis for these proactive attempts. Networking ability has been shown to influence human capital development behaviors and career opportunities and success (Seibert et al., 2001; Ng and Feldman, 2010) and to foster job performance and altruistic behaviors (Shi et al., 2011). Such benefits are obtained because networking ability helps build strong social ties and facilitates access to information and resources, all of which are central to performance (Sparrowe et al., 2001). Thus, a supervisor with high-networking ability is likely to exert significant power in the eyes of employees.

In sum, the above studies suggest that supervisor perceived organizational support, supervisor-organization value congruence, and supervisor networking all reflect the status of the supervisor in the organization, and as such should be positively related to, yet distinct from, PSSP:

*Hypothesis 2:* PSSP will be positively related to, yet distinct from perceptions of (a) supervisor networking ability, (b) organizational support directed at the supervisor, and (c) supervisor-organization value congruence.

## Perceived Supervisor Social Power Nomological Network

As noted by Bagozzi (1980), MacKenzie et al. (2011), when determining a new construct validity, it is not enough to examine its psychometric properties (i.e., factor structure and scale sore reliability) and its convergent and discriminant validity. They recommend that the nomological validity of the new construct be verified by integrating it into a theoretical model. Therefore, we specified the theoretical nature of the relations between PSSP and some of its outcomes. Based on PSSP influence mechanism, three propositions regarding its potential to influence followers were made to explore its nomological network : (a) PSSP should act as an antecedent of affective organizational commitment; (b) the relation between PSSP and turnover intention should be mediated by affective organizational commitment, (c) PSSP should positively moderate the relation between affective commitment to the supervisor and affective commitment to the organization.

### PSSP and Affective Organizational Commitment

As previously stated, PSSP theoretical influence mechanism proposes that as a result of an identification and internalization process of the supervisor’s power in the organization, employees come to see themselves as more powerful agents of the organization (Fiol et al., 2001) and to internalize organizational values and goals embodied by their supervisor (Ashforth et al., 2016). Affective commitment to the organization is an attitude held by followers that directly stems from their identification to the organizational goals and values (Meyer et al., 2004). Specifically, it is defined as an “emotional attachment to, identification with, and involvement in the organization” (Meyer and Allen, 1991, p. 67). Thus, we posit that it should be the primary and most proximal outcome of PSSP.

Two lines of research support the relation between PSSP and affective commitment to the organization. First, studies have shown that personal identification to the supervisor was positively related to employees’ affective commitment to the organization (van Vianen et al., 2011; Miao et al., 2012; Zhu et al., 2013). Furthermore, studies have shown that power bases stemming from the supervisor’s attributes and from subordinates’ identification to him or her, such as expert and referent power, were positively associated with followers’ affective commitment to the organization (Pierro et al., 2013; Haller et al., 2018). Thus, as employees come to identify with their supervisor seen as a powerful agent of the organization via PSSP, they should be more likely to internalize the organizational goals and values embodied by their supervisor, and to see themselves as committed to the organization. Therefore, we position PSSP as a source of social power likely to foster followers’ positive attitudes toward the organization:

*Hypothesis 3:* PSSP will be positively related to employees’ organizational commitment.

### PSSP, Organizational Commitment, and Turnover Intention

Arguably, because of the role ascribed to organizational commitment as a central psychological mechanism underpinning the effects of PSSP, our theoretical proposition also assumes that the effects of PSSP on outcomes located at the level of the employees will be mediated by their levels of affective commitment to the organization. As a further test of nomological network, we specifically posit that PSSP should decrease employee turnover intention as a result of their increased affective commitment to the organization.

As proposed by Solinger et al. (2008), organizational commitment, as any other attitude, possesses affective, cognitive, and behavioral elements (Ajzen, 2001). Consistently, Meyer and Herscovitch (2001) stated that “commitment is a force that binds an individual to a course of action of relevance to one or more targets” (p. 301). In other words, as the employee is “bound by his or her commitment” (Meyer and Herscovitch, 2001, p. 311), s/he feels an obligation of reciprocation (Lavelle et al., 2007). Thus, highly committed employees tend to become more positive actors in their organizations, for example by increasing their levels of performance and citizenship behaviors, and by intending to remain employed in this organization (Meyer et al., 2002).

In addition, as previously stated, affective commitment entails identifying with the organization (Meyer and Allen, 1991). Thus, according to social identity theory (Tajfel and Turner, 1979), when employees identify with their organization, they are likely to support it and be attracted to it. Consequently, they are less likely to intend to leave their organization. Furthermore, the role played by affective commitment in the prediction of turnover intention has been repeatedly supported (for meta-analyses on this relation, see Meyer et al., 2002; Harrison et al., 2006; for recent studies positing affective commitment toward the organization has a mediating variable between various supervisors’ behaviors and turnover intention, see Mathieu et al., 2016; Kim and Beehr, 2020).

Therefore, we hypothesize that PSSP has the potential to decrease followers’ intention to leave. However, this relation should be fully mediated by employees’ affective commitment to the organization.

*Hypothesis 4:* Organizational commitment will mediate the negative relation between PSSP and turnover intention.

### The Moderating Role of PSSP in the Relation Between Commitments Toward the Supervisor and the Organization

Although PSSP’s potential for influence comes from the supervisors, it should primarily lead to affective organizational commitment among followers, not to affective commitment to the supervisor. As stated above, PSSP’s influence process takes place outside of the supervisor-follower dyad and involves a process of identification with the supervisor’s role as an agent of the organization. However, we posit that affective commitment to the supervisor should be considered in PSSP’s nomological network.

Commitment to the supervisor has been shown to be an antecedent of affective commitment to the organization. Specifically, research has demonstrated that attachment to the supervisor (i.e., affective commitment to the supervisor) transfers to the organization (i.e., organizational commitment) because supervisors are often seen as agents acting on behalf of the organization (Vandenberghe and Bentein, 2009).

Relatedly, studies have shown that the extent to which the supervisor is perceived to represent the organization, or his or her centrality within the organization, moderates the relation between attitudes and perceptions directed towards the supervisor and those directed at the organization. For example, Eisenberger et al. (2002) found that employees’ perceptions of supervisors’ organizational status moderated the relation between perceived supervisor support and perceived organizational support. Likewise, Vandenberghe et al. (2017) found that high supervisor-organization value congruence resulted in a stronger relation between affective commitment to the supervisor and organizational commitment. In other words, because powerful supervisors have a greater ability to influence organizational decisions owing to their centrality in the organization, they more clearly endorse their role as representatives of the organization. In turn, this should help followers to more clearly see their supervisor as an agent of the organization, thus promoting the transfer from affective commitment to the supervisor to affective commitment to the organization. Therefore, we suggest that affective commitment to the supervisor will be more positively related to organizational commitment when supervisors are perceived as powerful agents:

*Hypothesis 5:* PSSP will moderate the relation between affective commitment to the supervisor and organizational commitment such that this relation will be is stronger (vs. weaker) when PSSP is high (vs. low).

Moreover, research has shown that affective commitment toward the organization acts as a mediating variable between affective commitment to the supervisor and turnover intention (Panaccio and Vandenberghe, 2011; Huyghebaert et al., 2019). Therefore, in line with previous hypotheses, the moderating effect of PSSP in the relation between affective commitment to the supervisor and the organization should have an indirect effect on turnover intention. Specifically, the indirect negative relation between affective commitment to the supervisor and turnover intention should be stronger at high levels of PSSP:

*Hypothesis 6:* PSSP will moderate the indirect negative relation between affective commitment to the supervisor and turnover intention through organizational commitment such that this indirect relation will be stronger (vs. weaker) when PSSP is high (vs. low).

## Overview of Studies

Based on MacKenzie et al. (2011) recommendations, the factor structure and psychometric properties of the PSSP scale, its convergent and discriminant validity, the assessment of its nomological network, and its cross-cultural validation were empirically examined in three studies. Specific to the cross-cultural validation, this research assessed the invariance of the measurement structure of the PSSP scale across respondents from France (French-speaking), Canada (French- and English-speaking), and Romania. These three countries were specifically chosen as they significantly differ not only in terms of language, but also in terms of power distance (i.e., the relative acceptance of unequal distribution of power in society; Hofstede, 1991; see also^1^), which is respectively intermediate (France), low (Canada), and high (Romania).

*Study 1* included two samples of university alumni occupying diverse jobs in the private or public sectors in France (*N* = 350) and Canada (*N* = 271) who completed the original French version of the PSSP items. This study first assessed the factor structure of the PSSP measure and its psychometric properties. Furthermore, it offered a first assessment of cross-cultural generalizability by examining the measurement invariance of the PSSP factor structure across these samples. Then it assessed the nomological network of PSSP by testing whether scores on this measure were positively related to affective organizational commitment (Hypothesis 3), and whether PSSP moderated the relation between affective commitment to the supervisor and the organization (Hypothesis 5). *Study 2* pursued similar objectives and was also conducted in Canada but focused on English-speaking respondents (*N* = 462). The English version of the items was developed using a classical translation back-translation procedure, where the items were translated to English and then back-translated to the original language to assure that they kept their original meaning throughout the translation (Brislin, 1970). This study replicated *Study 1* in assessing the factor structure and psychometric properties of the PSSP measure, its invariance across samples (English respondents vs. French respondents [*Study 1*, samples 1 and 2]), and its nomological network (Hypotheses 3 and 5). In addition, respondents also completed a measure of employees’ perceptions of their supervisor’s bases of power for purposes of assessing the convergent and discriminant validity of the PSSP scale (Hypothesis 1). Finally, Study 3 was conducted in Romania using a two-wave time-lagged design with a 2-month lag on a sample of employees from the private and public sectors (Time 1 *N* = 244, Time 2 *N* = 152). Specifically, PSSP and affective commitment to the supervisor (i.e., exogenous variables) were measured at Time 1, while affective commitment to the organization and turnover intentions (i.e., endogenous variables) were measured at Time 2. The same classical translation back-translation procedure from the original French version of the PSSP scale was used to create the Romanian version of PSSP items. This study also replicated *Study* 1 *and Study 2*. First, it assessed the factor structure, psychometric properties and invariance across countries (Romanian vs. French and Canadian [combined samples from *Study 1* and *Study 2*]) of the PSSP measure. Then, in a test of convergent and discriminant validity, *Study 3* assessed whether PSSP was related yet distinguishable from supervisor networking ability, perceptions of supervisor organizational support, and supervisor-organization value congruence (Hypothesis 2), all measured at Time 1. Finally, the nomological network of the PSSP measure was once again tested by assessing if affective organizational commitment measured at Time 2 mediated the relation between PSSP measured at Time 1 and turnover intention measured at Time 2 (Hypotheses 3 and 4). It further tested whether PSSP acted as a moderator in the mediational relation between affective commitment to the supervisor measured at Time 1 and the same measures of affective organizational commitment (Hypothesis 5) and turnover intention (Hypothesis 4).

## Development of the PSSP Scale

### Item Generation

Items were first generated based on the proposed unidimensional definition of PSSP. We thus ascertained that the initial pool of items covered all aspects of PSSP’s definition, while keeping in mind that the measure should remain as parsimonious as possible and unidimensional. More precisely, items were formulated to capture a single dimension referring to employees’ general perception of their supervisor’s influence. This perceived influence had to target other organizational agents or the organizational decision-making process *in general*, as employees are often not privy to the specifics of their supervisor’s social power toward targets other than themselves and their closest colleagues. Moreover, the item generation phase was driven by the concern that items should not tap into the power bases of the supervisor nor into employees’ commitment to the supervisor or to the organization. In so doing, our approach qualified as being deductive (Hinkin, 1998). This step of item generation resulted in an initial pool of 8 items that were then reviewed by three independent experts in the field of industrial and organizational psychology. Using the criteria mentioned above, these experts independently identified 5 of the 8 items as being correctly phrased and appropriate reflections of the PSSP construct.

### Expert Review of the Items

We then conducted an expert review of the content validity of the retained 5 items. A panel of 10 subject matter experts, all academic researchers (9 professors and 1 postdoctoral fellow) in the fields of industrial and organizational psychology or organizational behavior evaluated the items. PSSP’s definition was first presented to them. Next, they were asked to rate on as scale from 1 (totally disagree) to 5 (totally agree), to what extent they judged that the content of each item was representative of PSSP definition (Cai et al., 2017). It was decided prior to this expert review that an item with a mean score lower than 3/5 would be removed. As a further test of content validity, we also asked each expert to assess if each item was central to the measurement of the construct (Lawshe, 1975). Specifically, we asked experts to judge whether an item was “*essential*,” “useful, but not essential,” or “not necessary” (Zacher and Yang, 2016). Lawshe (1975) recommended that an item presents content validity if more than half of the experts rate it as essential. Results showed that no item presented a mean score of content adequacy lower than 3/5 (4.4 < *M* < 4.8). Furthermore, all items were deemed essential by more than half of the experts (Min: 70%, Max: 90%), and none of the experts judged an item unnecessary. We thus proceeded to the next steps of the psychometric assessment of the PSSP scale.

## Study 1 Method

### Participants and Procedure

#### Sample 1

We obtained the agreement of the alumni association of a business school located in France to conduct a study on job attitudes. In total, 1,765 individuals received a questionnaire and a consent form. They returned their responses using a pre-paid envelope. Among them, 350 provided usable responses (response rate = 19.83%). Respondents were affiliated with a variety of industries (e.g., consulting, banking, and transportation).

#### Sample 2

As for sample 1, we obtained agreement from the alumni association of a business school (located in Canada) to conduct a study on job attitudes. An announcement of the study was made on the website of the association and a link to the consent form and survey was provided. We obtained usable responses from 271 French speaking participants. Demographic characteristics for all samples are reported in Table 1. In this study, participants completed the questionnaire in French.

**TABLE 1:** Demographics by Sample.

	*Study 1*, Sample 1 France (*N* = 350)	*Study 1*, Sample 2 Canada-French (*N* = 271)	*Study 2* Canada-English (*N* = 462)	*Study 3* Romania T1 (*N* = 244)
Male (%)	53.7	49.6	56.3	30.0
Age (year): M (SD)	32.93 (6.82)	38.88 (10.61)	44.90 (10.58)	31.01 (8.61)
Organizational Tenure (year): M (SD)	5.23 (4.87)	8.61 (7.56)	11.32 (9.54)	3.96 (4.47)
Tenure with the Supervisor (year): M (SD)	2.64 (2.73)	4.10 (3.69)	4.24 (5.07)	2.61 (3.15)
Size of the Organization (> 1,000)	54.3%	43.7%	55.4%	8.0%
Private sector	-	66.8%	69.5%	78.6%

### Measures

The final five items of *PSSP* scale were measured and showed excellent scale score reliability (α_Sample 1_ = 0.925; α_Sample 2_ = 0.913). *Affective commitment to the supervisor* was measured with 6 items (α_Sample 1_ = 0.946; α_Sample 2_ = 0.955; e.g., “My supervisor means a lot to me”) from Vandenberghe and Bentein (2009). *Organizational commitment* was assessed with the French adaptation (Bentein et al., 2005) of Meyer et al.’s (1993) 6-item scale (α_Sample 1_ = 0.877; α_Sample 2_ = 0.902; e.g., “I really feel that I belong in this company”).

### Analyses

Analyses relied on Mplus 8 (Muthén and Muthén, 2017) robust weighted least square estimator (WLSMV) for ordered-categorical items with unequal response thresholds such as those used here (Finney and DiStefano, 2013). WLSMV is well-suited to initial development of measures across cultural contexts given its ability to closely reflect response thresholds (i.e., points at which responses move from one category to the other; Freund et al., 2013). Models were estimated using the full information available (Asparouhov and Muthén, 2010) to handle the few missing responses (Sample 1: 0 to 86%, *M* = 0.23%; Sample 2: 2.58% to 4.43%, *M* = 3.38%).

First, we assessed the factor structure of PSSP items by fitting a one-factor confirmatory factor analytic (CFA) model in each sample. Second, we assessed the cross validity or generalizability (i.e., lack of measurement bias) of this solution across samples through sequential tests of measurement invariance *(multigroup analysis)*, adapted to WLSMV (Millsap, 2011; Morin et al., 2011): (i) configural (i.e., same structure); (ii) weak (i.e., loadings); (iii) strong (i.e., loadings and thresholds); and (iv) strict (i.e., loadings, thresholds, and uniquenesses). Third, we assessed nomological network of PSSP by estimating Structural Equation Models (SEM; Bollen, 1989) including PSSP, affective commitment to the supervisor, and affective commitment to the organization. Before estimating these models, we first checked the invariance of the three-factor CFA model across samples and built the predictive model from the most invariant measurement model. In the predictive model, PSSP and affective commitment to the supervisor were specified as predictors of organizational commitment. We then assessed the equivalence of the regressions across samples in the following sequence: (i) regression slopes, (ii) regression intercepts, and (iii) regression disturbances (or regression residuals, reflecting the proportion of the variance in the outcome not explained by the predictors).

Finally, to test the moderating role of PSSP on the relation between affective commitment to the supervisor and organizational commitment, an interaction term was incorporated to the final SEM model. However, tests of latent interactions corrected for measurement error cannot be implemented using WLSMV, and tests of interactions are sensitive to measurement error (Marsh et al., 2013). To overcome this problem, we considered three approaches: (i) relying on single indicator latent variables defined by scale scores (i.e., the means of all items) corrected for measurement error based on a known estimate of reliability (Cheung and Lau, 2017); (ii) using factor scores from the measurement model including all constructs (Skrondal and Laake, 2001); (iii) using single indicator latent variables defined by factor scores corrected for measurement error based on a known estimate of reliability (Morin et al., 2020). Across all three studies, the results obtained using uncorrected factor scores proved most efficient at replicating the SEM results, hence was retained for tests of interactions. Tests of equivalence of these predictions across samples were conducted in the sequence described above, using robust maximum likelihood (MLR) estimation. In these tests, the initial unconstrained model is just identified (as any multiple regression model), and thus will always achieve perfect fit to the data. However, subsequent models gain degrees of freedom and can be contrasted with this initial model.

To account for the oversensitivity of the chi-square test to minor model misspecifications to sample size variations (Marsh et al., 2005), we additionally used the following indices: the comparative fit index (CFI), the Tucker-Lewis index (TLI), and the root mean square error of approximation (RMSEA) with its 90% confidence interval (CI). Values greater than 0.900 and 0.950 for the CFI and TLI, respectively, indicate adequate and excellent fit, while RMSEA values smaller than 0.100, 0.080, and 0.060 indicate acceptable, good, and excellent fit, respectively (Yu, 2002). In comparing nested models, models differing by less than.010 on the CFI and TLI, or 0.015 on the RMSEA, can be considered to provide equivalent fit to the data (Chen, 2007). For all models, we reported model-based omega (ω) coefficients of composite reliability (McDonald, 1970).

## Study 1 Results

### PSSP Factor Structure and Invariance Across Samples

Fit indices for the PSSP CFA models are reported in Table 2. PSSP unidimensional factor structure was empirically supported as it presented a good fit to the data in both samples according to RMSEA and an excellent fit according to CFI and TLI. Parameter estimates (i.e., factor loadings and uniquenesses) are reported in Table 3 and reveal strong factor loadings on the PSSP factor and satisfactory estimates of composite reliability (ω_sample 1_ = 0.947; ω_sample 2_ = 0.938). Tests of measurement invariance confirmed the generalizability of this solution across samples as none of the constraints resulted in a decrease in fit exceeding the recommended guidelines.

**TABLE 2:** Fit Indices for the Models Estimated in Study 1.

Model	χ^2^ (*df*)	CFI	TLI	RMSEA	90% CI	Δ χ^2^ (Δ *df*)	Δ CFI	Δ TLI	ΔRMSEA
*PSSP measurement model*									
Sample 1 (France)	14.650 (5)	0.999	0.998	0.074	0.032; 0.120	−	−	−	−
Sample 2 (Canada)	15.724 (5)^∗^	0.999	0.998	0.090	0.042; 0.142	−	−	−	−
*PSSP measurement invariance*									
Configural invariance	30.403 (10)^∗^	0.999	0.998	0.082	0.049; 0.116	−	−	−	−
Weak invariance	35.179 (14)^∗^	0.999	0.999	0.070	0.042; 0.100	9.774 (4)	0.000	+0.001	−0.012
Strong invariance	90.016 (28)^∗^	0.997	0.998	0.084	0.066; 0.105	58.004 (14)^∗^	−0.002	+0.001	+0.014
Strict invariance	121.122 (33)^∗^	0.996	0.997	0.093	0.076; 0.111	31.453 (5)^∗^	−0.001	−0.002	+0.009
*Full measurement model invariance across samples*									
Configural invariance	804.867 (232)^∗^	0.984	0.981	0.090	0.083; 0.096	−	−	−	−
Weak invariance	828.243 (246)^∗^	0.984	0.982	0.088	0.081; 0.094	28.366 (14)^∗^	0.000	+0.001	−0.002
Strong invariance	932.943 (294)^∗^	0.982	0.983	0.084	0.078; 0.090	130.371 (48)^∗^	−0.002	+0.001	−0.004
Strict invariance	1019.530 (311)^∗^	0.980	0.983	0.086	0.080; 0.092	111.148 (17)^∗^	−0.002	0.000	+0.002
*Predictive validity*									
Predictive model	1019.528 (311)^∗^	0.980	0.983	0.086	0.080; 0.092	−	−	−	−
Predictive model – Invariant slopes	769.341 (313)^∗^	0.987	0.989	0.069	0.063; 0.075	1.921 (2)	+0.007	+0.006	−0.017
Predictive model – Invariant intercepts	748.828 (314)^∗^	0.988	0.989	0.067	0.061; 0.073	0.697 (1)	+0.001	0.000	−0.002
Predictive model – Invariant disturbances	787.854 (315)^∗^	0.987	0.988	0.070	0.064; 0.076	10.926 (1)	−0.001	−0.001	+0.003
*Moderation*									
Moderated Predictive Model (just identified)	0 (0)	1.000	1.000	0.000	0.000; 0.000	−	−	−	−
Moderated Predictive Model – Invariant slopes	2.174 (3)	1.000	1.004	0.000	0.000; 0.093	1.929 (3)	0.000	+0.004	0.000
Moderated Predictive Model – Invariant intercepts	4.179 (4)	0.999	0.998	0.011	0.000; 0.088	1.602 (1)	−0.001	−0.006	+0.011
Moderated Predictive Model – Invariant disturbances	26.378 (5)^∗^	0.864	0.837	0.118	0.076; 0.164	43.768 (1)^∗^	−0.135	−0.161	+0.107

*PSSP = perceived supervisor social power; χ^2^ = chi-square test of exact fit; *df* = degrees of freedom; CFI = comparative fit index; TLI = Tucker-Lewis index; RMSEA = root mean square error of approximation; CI = confidence interval for the RMSEA; Δ = change in fit relative to the preceding model. Chi-square difference tests were calculated using Mplus DIFFTEST function for WLSMV estimation (Asparouhov and Muthén, 2006) for most models reported here, except for the moderation models (estimated with MLR) which were calculated using the Satorra-Bentler correction (Satorra, 2000). ^∗^*p* < 0.01.*

**TABLE 3:** Items and Standardized Parameter Estimates for the PSSP Scale.

				*Study 1*, Sample 1 (France)		*Study 1*, Sample 2 (French Canada)		*Study 2* (English Canada)		*Study 3* (Romania)	
	French Item	English Item	Romanian Item	λ	δ	λ	δ	λ	δ	λ	δ
1	Mon supérieur est écouté dans cette entreprise	People listen to my supervisor in this company	Seful meu este ascultat in aceasta companie	0.675	0.544	0.650	0.578	0.555	0.692	0.664	0.559
2	La position de mon supérieur dans l’entreprise lui confère beaucoup de pouvoir	My supervisor’s position in this company gives him/her a lot of power	Pozitia ocupata de seful meu ii confera acestuia multa putere	0.945	0.107	0.967	0.064	0.863	0.255	0.919	0.155
3	Les décisions de mon supérieur touchent beaucoup de monde dans cette entreprise	My supervisor’s decisions affect a lot of people in this company	Deciziile sefului meu au o influenta asupra multor persoane in aceasta companie	0.927	0.141	0.875	0.234	0.822	0.324	0.886	0.214
4	Mon supérieur fait partie du cercle des gens les plus influents dans cette entreprise	My supervisor is part of the inner circle of most influential people in this company	Seful meu face parte din cercul oamenilor celor mai influenti din aceasta companie	0.947	0.104	0.960	0.078	0.873	0.238	0.897	0.195
5	Par rapport à ses collègues, mon supérieur dispose d’une marge de manoeuvre importante dans l’entreprise	Compared to his colleagues, my supervisor has an important freedom of action in this company	Prin comparatie cu colegii sai, seful meu dispune de o marja de manevra importanta in aceasta companie	0.898	0.193	0.854	0.270	0.844	0.288	0.873	0.237
ω				0.947		0.938		0.897		0.930	

*λ: factor loading (all factors loadings are higher than 0.500 and thus can be considered to be adequate; Kline, 2016); δ: item uniqueness; ω: omega coefficient of model-based composite reliability (McDonald, 1970), ω = (Σ| λ_i_|)^2^/([Σ| λ_i_|]^2^+Σδ_ii_). Responses were provided on a scale ranging from 1 (French: *pas du tout d’accord*; English: *completely disagree*; Romanian: *dezacord total*) to 5 (French: *tout à fait d’accord*; English: *completely agree*; Romanian: *total de acord*).*

### PSSP Nomological Network

As shown in Table 2, the CFA model encompassing all constructs (i.e., PSSP, affective commitment to the supervisor, and affective commitment to the organization) yielded a good fit and was fully invariant across samples. Latent variable correlations from this model and estimates of composite reliability are reported in the top section of Table 4. All factors were defined by satisfactory factor loadings and estimates of composite reliability (ω = 0.907 to 0.974 across constructs and samples). This CFA model was then converted to our *a priori* SEM model. Results (Table 2) indicated that the structural paths were fully equivalent across samples as none of the equality constraints resulted in a decrease in fit exceeding the recommended guidelines. Predictive coefficients obtained as part of this predictive model are reported in Table 5 and show that PSSP was positively related to organizational commitment (Sample 1: β = 0.197, *p* < 0.01, Sample 2: β = 0.198, *p* < 0.01), supporting Hypothesis 3.

**TABLE 4:** Latent Variable Correlations and Composite Reliabilities for Variables across Samples and Studies.

*Study 1*: Sample 1	PSSP	Organizational commitment	Affective commitment to the supervisor					
PSSP								
Organizational commitment	0.419^∗∗^							
Affective commitment to the supervisor	0.460^∗∗^	0.463^∗∗^						
Reliability (ω)	0.948	0.907	0.967					

***Study 1*: Sample 2**	**PSP**	**Organizational commitment**	**Affective commitment to the supervisor**					

PSSP								
Organizational commitment	0.259^∗∗^							
Affective commitment to the supervisor	0.272^∗∗^	0.456^∗∗^						
Reliability (ω)	0.951	0.937	0.974					

***Study 2***	**PSSP**	**Organizational commitment**	**Affective commitment to the supervisor**	**Reward power**	**Coercive power**	**Legitimate power**	**Expert power**	**Referent power**

PSSP								
Organizational commitment	0.414^∗∗^							
Affective commitment to the supervisor	0.520^∗∗^	0.630^∗∗^						
Reward power	0.402^∗∗^	0.054	0.083					
Coercive power	−0.154^∗∗^	−0.365^∗∗^	−0.496^∗∗^	0.196^∗∗^				
Legitimate power	0.320^∗∗^	0.105^∗^	0.124^∗∗^	0.359^∗∗^	0.449^∗∗^			
Expert power	0.448^∗∗^	0.380^∗∗^	0.636^∗∗^	0.301^∗∗^	−0.187^∗∗^	0.372^∗∗^		
Referent power	0.385^∗∗^	0.476^∗∗^	0.712^∗∗^	0.162^∗∗^	−0.200^∗∗^	0.384^∗∗^	0.722^∗∗^	
Reliability (ω)	0.921	0.950	0.985	0.923	0.957	0.948	0.930	0.977

***Study 3***	**PSSP**	**Organizational commitment**	**Affective commitment to the supervisor**	**Turnover intention**	**Supervisor networking ability**	**Supervisor POS**	**S−O value congruence**	

PSSP								
Organizational commitment	0.331^∗∗^							
Affective commitment to the supervisor	0.448^∗∗^	0.279^∗∗^						
Turnover intention	−0.302^∗∗^	−0.302^∗∗^	−0.302^∗∗^					
Supervisor networking ability	0.676^∗∗^	0.237^∗∗^	0.536^∗∗^	−0.139				
Supervisor POS	0.621^∗∗^	0.435^∗∗^	0.465^∗∗^	−0.268^∗∗^	0.494^∗∗^			
S-O value congruence	0.587^∗∗^	0.410^∗∗^	0.657^∗∗^	−0.289^∗∗^	0.603^∗∗^	0.590^∗∗^		
Reliability (ω)	0.945	0.941	0.985	0.960	0.951	0.944	0.960	

*PSSP = perceived supervisor social power; POS = perceived organizational support; S-O = supervisor-organization; ω = omega coefficient of composite reliability. ^∗^*p* < 0.05; ^∗∗^*p* < 0.01.*

**TABLE 5:** Predictive Model Results across Studies and Samples.

	Study 1, Sample 1: Organizational commitment		Study 1, Sample 2: Organizational commitment		Study 2: Organizational commitment		Study 3: Organizational commitment (Time 2)		Study 3: Turnover intention (Time 2)	
	b (s.e.)	β (s.e.)	b (s.e.)	β (s.e.)	b (s.e.)	β (s.e.)	b (s.e.)	β (s.e.)	b (s.e.)	β (s.e.)
*Predictive Models*										
PSSP	0.394 (0.087)^∗∗^	0.197 (0.039)^∗∗^	0.394 (0.087)^∗∗^	0.198 (0.040)^∗∗^	0.091 (0.046)^∗^	0.155 (0.046)^∗^	0.083 (0.149)	0.073 (0.131)	−0.252 (0.119)^∗^	−0.265 (0.131)^∗^
Affective commitment to the supervisor	0.459 (0.058)^∗∗^	0.364 (0.036)^∗∗^	0.459 (0.058)^∗∗^	0.421 (0.042)^∗∗^	0.258 (0.038)^∗∗^	0.462 (0.063)^∗∗^	−0.003 (0.126)	−0.002 (0.111)	−0.300 (0.152)^∗^	−0.223 (0.112)^∗^
Reward power					−0.030 (0.041)	−0.032 (0.044)				
Coercive power					−0.176 (0.081)^∗^	−0.127 (0.057)^∗^				
Legitimate power					0.060 (0.057)	0.058 (0.055)				
Expert power					−0.146 (0.074)^∗^	−0.111 (0.056)^∗^				
Referent power					0.090 (0.047)	0.126 (0.065)				
Supervisor networking ability							−0.132 (0.128)	−0.116 (0.112)	0.260 (0.146)	0.230 (0.125)
Supervisor POS							0.330 (0.138)^∗^	0.289 (0.114)^∗^	0.024 (0.128)	0.021 (0.113)
S-O value congruence							0.306 (0.134)^∗^	0.268 (0.112)^∗^	−0.030 (0.152)	−0.026 (0.135)
Organizational commitment									−0.271 (0.079)	−0.273 (0.075)^∗∗^
*R*^2^	0.238 (0.032)^∗∗^		0.260 (0.034)^∗∗^		0.424 (0.033)^∗∗^		0.232 (0.062)^∗∗^		0.219 (0.058)^∗∗^	

	**Study 1, Sample 1: Organizational commitment**		**Study 1, Sample 2: Organizational commitment**		**Study 2: Organizational commitment**		**Study 3: Organizational commitment (Time 2)**		**Study 3: Turnover intention (Time 2)**	
	**b (s.e.)**	**β (s.e.)**	**b (s.e.)**	**β (s.e.)**	**b (s.e.)**	**β (s.e.)**	**b (s.e.)**	**β (s.e.)**	**b (s.e.)**	**β (s.e.)**

*Moderation Models*										
PSSP	0.229 (0.036)^∗∗^	0.266 (0.041)^∗∗^	0.229 (0.036)^∗∗^	0.227 (0.036)^∗∗^	0.204 (0.061)^∗^	0.189 (0.057)^∗^	0.030 (0.082)	0.033 (0.091)	−0.325 (0.074)^∗∗^	−0.306 (0.078)^∗∗^
Affective commitment to the supervisor	0.397 (0.041)^∗∗^	0.444 (0.044)^∗∗^	0.397 (0.041)^∗∗^	0.399 (0.042)^∗∗^	0.450 (0.077)^∗∗^	0.444 (0.074)^∗∗^	0.046 (0.078)	0.053 (0.091)	−0.238 (0.061)^∗∗^	−0.404 (0.094)^∗∗^
PSSP^∗^Affective commitment to the supervisor	0.134 (0.035)^∗∗^	0.163 (0.045)^∗∗^	0.134 (0.035)^∗∗^	0.140 (0.038)^∗∗^	0.090 (0.010)^∗∗^	0.094 (0.006)^∗∗^	0.076 (0.032)^∗^	0.088 (0.037)^∗^	0.008 (0.033)	0.010 (0.042)
Reward power					−0.061 (0.047)	−0.059 (0.045)				
Coercive power					−0.134 (0.060)^∗^	−0.133 (0.059)^∗^				
Legitimate power					0.061 (0.066)	0.060 (0.065)				
Expert power					−0.129 (0.066)^∗^	−0.125 (0.064)^∗^				
Referent power					0.149 (0.076)^∗^	0.145 (0.073)^∗^				
Supervisor networking ability							−0.096 (0.064)	−0.115 (0.077)	0.269 (0.064)^∗∗^	0.358 (0.085)^∗∗^
Supervisor POS							0.282 (0.070)^∗∗^	0.335 (0.085)^∗∗^	0.031 (0.062)	0.041 (0.081)
S-O value congruence							0.267 (0.074)^∗∗^	0.321 (0.085)^∗∗^	0.003 (0.075)	0.004 (0.101)
Organizational commitment									−0.261 (0.078)^∗∗^	−0.291 (0.084)^∗∗^
*R*^2^	0.328 (0.039)^∗∗^		0.266 (0.037)^∗∗^		0.469 (0.039)^∗∗^		0.350 (0.056)^∗∗^		0.346 (0.034)^∗∗^	

*b = unstandardized regression coefficient; s.e. = standard error; β = standardized regression coefficient; PSSP = perceived supervisor social power; POS = perceived organizational support; S-O = supervisor-organization; ^∗^*p* < 0.05; ^∗∗^*p* < 0.01.*

**FIGURE 1:**
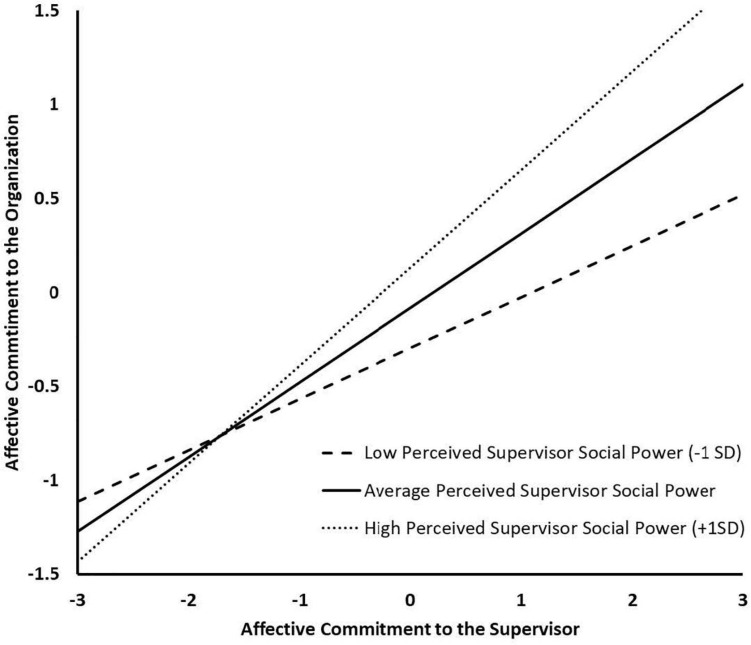
Study 1 Simple Slopes for the Moderation Effect of Perceived Supervisor Social Power between Affective Commitment to the Supervisor and Organizational Commitment.

The results associated with the final set of SEMs aimed at testing moderation (Fit indices: bottom of Table 2; coefficients: bottom of Table 5) supported the equivalence of the regression slopes and intercepts, but not disturbances (i.e., the regression residual), across samples. The absence of equivalent disturbances indicated that the percentage of explained variance in organizational commitment (R^2^) differed across samples. Specifically, this final model explained 26.6% (Sample 2) to 32.8% (Sample 1) of the variance in organizational commitment, suggesting that PSSP and commitment to the supervisor were slightly stronger predictors of organizational commitment among French workers. Importantly, this model revealed a significant interaction between PSSP and affective commitment to the supervisor in predicting organizational commitment (Sample 1: β = 0.163, *p* < 0.01, Sample 2: β = 0.140, *p* < 0.01). Simple slopes for the effect of affective commitment to the supervisor on organizational commitment at high (+1*SD*: *b* = 0.522, *SE* = 0.052, *p* < 0.01), moderate (*M*: *b* = 0.397, *SE* = 0.041, *p* < 0.01), and low (-1*SD*: *b* = 0.272, *SE* = 0.053, *p* < 0.01) levels of PSSP are depicted in Figure 1 and show that the positive effect of affective commitment to the supervisor increased linearly as a function of increases in PSSP, thus supporting Hypothesis 5.

## Study 2 Method

### Participants and Procedure

As part of a larger project, survey participants for *Study 2* were recruited through Legerweb, a Canadian web panel that includes 400 000 members across Canada. Recent research suggests that data collected through similar platforms are reliable and of good quality (e.g., Cheung et al., 2017). Participants received an email invitation asking them to participate in a survey of job attitudes. Participation was deemed to be voluntary and confidentiality of responses was ensured. Among 918 participants who were contacted, usable responses were obtained from 507 individuals. Excluding speedy respondents (*n* = 7; responding time < 1/3 median time) and careless respondents (*n* = 38; i.e., those who failed to tick the right answer to a trap item included in the survey), there remained 462 participants for purposes of the analyses (*M*_*age*_ = 44.90; 56.3% men). Sample characteristics are presented in Table 1. In this study, participants completed the English version of the questionnaire.

### Measures

Perceived supervisor social power (α = 0.862), affective commitment to the supervisor (α = 0.975), and organizational commitment (α = 0.931) were measured using the same scales as in *Study 1*. Respondents’ perceptions of the bases of their supervisor’s power were assessed using a 20-item measure created by Hinkin and Schriesheim (1989) and encompassing five four-item subscales: (a) reward power (α = 0.879; e.g., “My supervisor can increase my pay level”); (b) coercive power (α = 0.943; e.g., “My supervisor can make my work difficult for me”); (c) legitimate power (α = 0.910; e.g., “My supervisor can make me feel that I have commitments to meet”); (d) expert power (α = 0.900; e.g., “My supervisor can give me good technical suggestions”); and (e) referent power (α = 0.957; e.g., “My supervisor can make me feel important”).

### Analyses

Analyses were similar to those conducted in *Study 1*, although initial tests of measurement invariance for the PSSP measure were conducted across samples of French (*Study 1* Samples 1 and 2) vs. English (*Study 2*) respondents. In addition, this study aimed at assessing the convergent and discriminant validity of PSSP with respect to supervisors’ bases of power (French and Raven, 1959). First latent correlations among all variables considered in *Study 2* were computed to assess PSSP’s convergent validity. Then, as recommended by MacKenzie et al. (2011) discriminant validity was assessed by contrasting a CFA model where all constructs were freely correlated with one another to a series of more parsimonious models in which the correlation between PSSP and each basis of power was alternatively set to 1.0. These nested model comparisons were made using the same criteria as those used in *Study 1* (Chen, 2007). Finally, for tests of mediation and moderation, the bases of power were included together with PSSP and affective commitment to the supervisor as predictors of organizational commitment. In this study, due to the way the online testing platform was programmed, there were no missing responses.

## Study 2 Results

### PSSP Factor Structure and Measurement Invariance Across Studies

Fit indices for the models assessing the measurement invariance of the PSSP measure across studies are reported in Table 6. The results support the adequacy of the one-factor PSSP CFA model and its complete invariance across *Study 1* and *Study 2*. Parameter estimates are reported in Table 3 and reveal a PSSP factor defined by satisfactory factor loadings and composite reliability (ω = 0.897).

**TABLE 6:** Fit Indices for the Models Estimated in Study 2.

Model	χ^2^ (*df*)	CFI	TLI	RMSEA	90% CI	Δ χ^2^ (*df*)	Δ CFI	Δ TLI	Δ RMSEA
*PSSP Measurement Invariance across Studies (1 vs. 2)*									
Configural invariance	66.704 (10)*	0.998	0.995	0.103	0.080; 0.127	−	−	−	−
Weak invariance	62.301 (14)*	0.998	0.997	0.080	0.060; 0.101	11.873 (4)*	0.000	+0.002	−0.023
Strong invariance	95.587 (28)*	0.997	0.998	0.067	0.053; 0.082	41.548 (14)*	−0.001	+0.001	−0.013
Strict invariance	112.134 (33)*	0.997	0.998	0.067	0.053; 0.081	19.903 (5)*	0.000	0.000	0.000
*Discriminant Validity*									
Complete measurement model	2798.329 (601)*	0.974	0.971	0.089	0.086; 0.092	−	−	−	−
Constrained Model (PSSP = Reward Power)	4179.139 (602)*	0.958	0.953	0.113	0.110; 0.117	261.520 (1)*	−0.016	−0.018	+0.024
Constrained Model (PSSP = Coercive Power)	7969.478 (602)*	0.913	0.904	0.163	0.160; 0.166	654.194 (1)*	−0.061	−0.067	+0.074
Constrained Model (PSSP = Legitimate Power)	4685.724 (602)*	0.952	0.947	0.121	0.118; 0.124	299.849 (1)*	−0.022	−0.024	+0.032
Constrained Model (PSSP = Expert Power)	4225.405 (602)*	0.957	0.953	0.114	0.111; 0.117	304.147 (1)*	−0.017	−0.018	+0.025
Constrained Model (PSSP = Referent Power)	4864.027 (602)*	0.950	0.944	0.124	0.121; 0.127	329.725 (1)*	−0.024	−0.026	+0.035
*Predictive Validity*									
Complete predictive model	2801.371 (601)*	0.974	0.971	0.089	0.086; 0.092	−	−	−	−

*PSSP = perceived supervisor social power; χ^2^ = chi-square test of exact fit; *df* = degrees of freedom; CFI = comparative fit index; TLI = Tucker-Lewis index; RMSEA = root mean square error of approximation; CI = confidence interval for the RMSEA; Δ = change in fit relative to the preceding model. Chi-square difference tests were calculated using Mplus DIFFTEST function for WLSMV estimation (Asparouhov and Muthén, 2006). ^∗^*p* < 0.01.*

### Convergent and Discriminant Validity

The unconstrained (theorized) CFA model including all variables considered in this study yielded an excellent fit to the data according to the CFI and TLI and an acceptable fit according to the RMSEA (see Table 6, Unconstrained model). Latent variable correlations for this model and estimates of composite reliability are reported in Table 4. All factors were defined by satisfactory factor loadings and estimates of composite reliability (ω = 0.921 to 0.985), and correlations supported the relation between PSSP and the various bases of power (*r* = −0.154 to 0.448). None of these correlations were high enough to suggest construct redundancy or a problematic level of overlap (Kline, 2016). Results for the tests of discriminant validity are reported in Table 6. Each of the constrained models (where the correlation between PSSP and each of the power bases was alternatively fixed to 1.0) resulted in a decrease in model fit according to all indices that exceeded the recommended criteria in comparing nested models (i.e., indicating a difference between models; Chen, 2007). These results indicate that, although related to the power bases, PSSP taps into a distinct construct domain, supporting Hypothesis 1.

### PSSP Nomological Network

**FIGURE 2:**
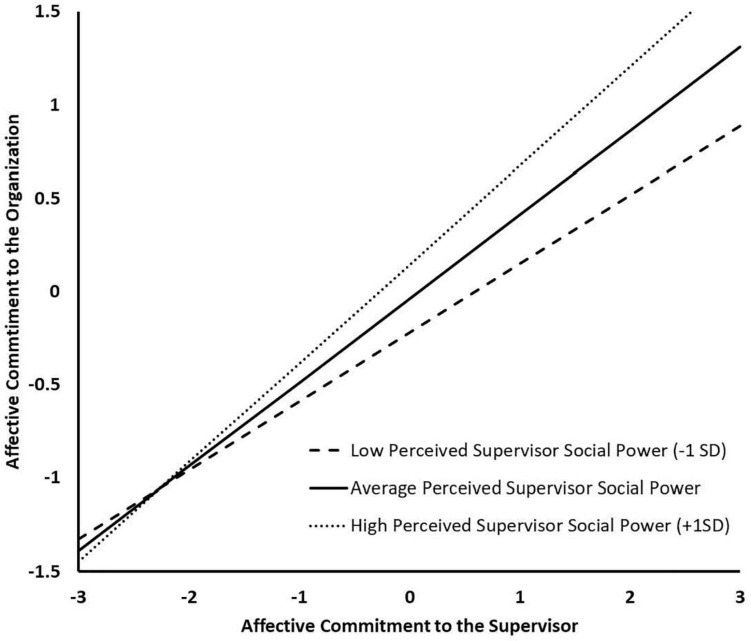
Study 2 Simple Slopes for the Moderation Effect of Perceived Supervisor Social Power between Affective Commitment to the Supervisor and Organizational Commitment.

The unconstrained CFA model was then converted to our *a priori* SEM model. This model achieved a satisfactory fit to the data (see Table 6). Structural paths associated with this predictive model are reported in Table 5. The results show that, over and above the effects of the bases of power, PSSP was positively related to organizational commitment (β = 0.155, *p* < 0.05), yielding support for Hypothesis 3. Among power bases, coercive and expert power were negatively related to organizational commitment (β = −0.127, *p* < 0.05, and β = −0.111, *p* < 0.05, respectively). Furthermore, in the final moderated model, PSSP interacted significantly with affective commitment to the supervisor to predict organizational commitment (β = 0.094, *p* < 0.01; bottom section of Table 5). Simple slopes representing the effects of affective commitment to the supervisor on organizational commitment at high (+1*SD*: *b* = 0.530, *SE* = 0.077, *p* < 0.01), moderate (*M*: *b* = 0.450, *SE* = 0.077, *p* < 0.01), and low (−1*SD*: *b* = 0.369, *SE* = 0.077, *p* < 0.01) levels of PSSP are depicted in Figure 2 and show that the positive effect of affective commitment to the supervisor increased linearly as a function of increases in PSSP, thus supporting Hypothesis 5. This final model explained 46.9% of the variance in organizational commitment. Importantly, these predictive results are similar to those obtained in *Study 1*, showing that controlling for the bases of power does little to modify these relations.

## Study 3 Method

### Participants and Procedure

Data for *Study 3* was collected through convenience sampling from Romanian employees affiliated with public and private firms. Participants received an email describing the study and a link to the questionnaire. They were advised that the study included two waves separated by 2 months. A Romanian version of the items was developed using a classical translation back-translation procedure. We measured PSSP, supervisor networking ability, supervisor perceived organizational support, supervisor-organization value congruence, and affective commitment to the supervisor at Time 1, and organizational commitment and turnover intention at Time 2. Usable responses were obtained from 244 participants at Time 1 (*M* age = 31.01; 30.0% men) and 152 at Time 2. Sample characteristics are presented in Table 1.

### Measures

Perceived supervisor social power (α = 0.902), affective commitment to the supervisor (α = 0.967), and organizational commitment (α = 0.918) were measured using the same scales as in *Study 1* and *Study 2*. *Supervisor networking ability* was assessed through a 6-item scale (α = 0.928; e.g., “My supervisor spends a lot of time and effort at work networking with others”) developed by Ferris et al. (2005). *Supervisor perceived organizational support* was measured using a reduced 8-item version (e.g., Vandenberghe et al., 2004) of Eisenberger et al.’s (1986) perceived organizational support survey in which items were adapted to reflect support from the organization to the supervisor (α = 0.924; e.g., “My organization really cares about my supervisor’s well-being”). *Supervisor-organization value congruence* was measured using Vandenberghe et al.’s (2017) 6-item scale (α = 0.939; e.g., “My supervisor shares this organization’s values”). Finally, *turnover intention* was measured by three items (α = 0.936; e.g., “I often think about quitting this organization”) adapted from Hom and Griffeth (1991) and Jaros (1997).

### Analyses

Analyses replicated those used in *Study 1* and *Study 2*, with the following caveats: (a) initial tests of measurement invariance were conducted across samples of Romanian (Study 3) vs. French and Canadian (combined samples from *Study 1* and *Study 2*) respondents; (b) the SEM model contained PSSP, affective commitment to the supervisor, supervisor networking ability, supervisor perceived organizational support, and supervisor-organization value congruence as Time 1 predictors of Time 2 organizational commitment and turnover intention; (c) convergent and discriminant validity was examined with respect to supervisor networking ability, supervisor POS, and supervisor-organization value congruence; (d) missing data was minimal among participants who completed the time specific measures (Time 1: 0% to 2.05%, *M* = 0.77%; Time 2: 0% to 0.66%, *M* = 0.15%) and all models were estimated using available information from participants who completed at least one measurement point (*N* = 244; Asparouhov and Muthén, 2010). Finally, to test Hypothesis 4, or *a priori* model of full mediation (in which affective commitment to the supervisor, supervisor networking ability, supervisor POS, and supervisor-organization value congruence were specified as predictors of organizational commitment which in turn was specified as a predictor of turnover intention) was contrasted with a model of partial mediation (adding direct links between the predictors and the outcomes) to verify whether the mediation was indeed complete, or whether additional direct effects remained (MacKinnon, 2008; Hayes, 2013). These analyses were conducted using predictive SEM models and revealed a lack of association between PSSP and affective commitment but moderately strong direct effects of the predictors on the outcomes, hence the model of partial mediation was retained. In this model, the statistical significance of the indirect effect of PSSP on turnover intention through organizational commitment was calculated with a 95% bias-corrected bootstrap (based on 10,000 bootstrap samples) CI (Cheung and Lau, 2008).

## Study 3 Results

### PSSP Factor Structure and Invariance Across Studies

Fit indices for the models assessing the measurement invariance of the PSSP measure across studies are reported in Table 7. The results supported for a third time the adequacy of the one-factor PSSP CFA model and its complete invariance across the combined samples used in *Study 1* and *Study 2* vs. *Study 3*. Parameter estimates are reported in Table 3 and reveal a PSSP factor defined by satisfactory factor loadings and composite reliability (ω = 0.930).

**TABLE 7:** Fit Indices for the Models Estimated in Study 3.

Model	χ^2^ (*df*)	CFI	TLI	RMSEA	90% CI	Δ χ^2^ (*df*)	Δ CFI	Δ TLI	Δ RMSEA
*PSSP Measurement Invariance Across Studies*									
Configural invariance	65.028 (10)*	0.998	0.996	0.091	0.071; 0.113	−	−	−	−
Weak invariance	84.302 (14)*	0.997	0.996	0.087	0.070; 0.106	24.576 (4)*	−0.001	0.000	−0.004
Strong invariance	134.897 (82)*	0.996	0.997	0.076	0.063; 0.089	64.346 (14)*	−0.001	+0.001	−0.011
Strict invariance	203.456 (33)*	0.993	0.996	0.088	0.077; 0.100	70.307 (5)*	−0.003	−0.001	+0.012
*Discriminant Validity*									
Unconstrained model	1529.725 (719)*	0.974	0.972	0.068	0.063; 0.073	−	−	−	−
Constrained Model (PSSP = Supervisor networking ability)	1925.993 (720)*	0.961	0.958	0.083	0.078; 0.087	95.362 (1)*	−0.013	−0.014	+0.015
Constrained Model (PSSP = S-O value congruence)	217.660 (720)*	0.954	0.950	0.090	0.086; 0.095	119.573 (1)*	−0.020	−0.022	+0.022
Constrained Model (PSSP = Supervisor POS)	2069.717 (720)*	0.957	0.953	0.088	0.083; 0.092	104.139 (1)*	−0.017	−0.019	+0.020
*Predictive Validity*									
Complete predictive model	1529.725 (719)*	0.974	0.972	0.068	0.063; 0.073	−	−	−	−

*PSSP = perceived supervisor social power; χ^2^ = chi-square test of exact fit; *df* = degrees of freedom; CFI = comparative fit index; TLI = Tucker-Lewis index; RMSEA = root mean square error of approximation; CI = confidence interval for the RMSEA; Δ = change in fit relative to the preceding model; S-O = supervisor-organization; POS = perceived organizational support. Chi-square difference tests were calculated using Mplus DIFFTEST function for WLSMV estimation (Asparouhov and Muthén, 2006). ^∗^*p* < 0.01.*

### Convergent and Discriminant Validity

The unconstrained CFA model including all variables considered in this study yielded an excellent fit according to the CFI and TLI and an acceptable fit according to the RMSEA (see Table 7, Unconstrained model). Latent variable correlations from this model and estimates of composite reliability are reported in Table 4. All factors were defined by satisfactory factor loadings and estimates of composite reliability (ω = 0.941 to 0.985), and correlations supported the relation between PSSP and supervisors’ networking ability (*r* = 0.676), supervisor perceived organizational support (*r* = 0.621), and supervisor-organization value congruence (*r* = 0.587). Once again, none of these correlations were high enough to suggest construct redundancy or a problematic level of overlap (Kline, 2016). Results of the tests for discriminant validity are reported in Table 7. Each of the constrained CFA models (where the correlation between PSSP and each of the covariates was alternatively set to 1.0) resulted in a decrease in model fit according to all indices, that once again exceeded the suggested criteria for nested model comparison (Chen, 2007). These results indicate that, although related to these constructs, PSSP taps into a distinct construct domain, thereby supporting Hypothesis 2.

### PSSP Nomological Network

The SEM predictive model yielded a satisfactory fit according to all indices (see Table 7)^2^. The parameter estimates for this model are reported in Table 5, and reveal that controlling for Time 1 supervisor networking ability, supervisor POS, and supervisor-organization value congruence, Time 1 PSSP was unrelated to Time 2 organizational commitment (β = 0.073, *ns*), which contradicts Hypothesis 3. Interestingly, affective commitment to the supervisor was also unrelated to organizational commitment at Time 2 (β = −0.002, *ns*). Of further interest, over and above the effect of organizational commitment (β = −0.273, *p* < 0.01), PSSP and affective commitment to the supervisor were both negatively associated with Time 2 turnover intention (β = −0.265, *p* < 0.05; and β = −0.223, *p* < 0.05, respectively). The indirect effect of PSSP on turnover intention through organizational commitment was non-significant (indirect effect = −0.023, *SE* = 0.041, 95% bootstrap CI = −0.150, 0.062). Thus, Hypothesis 4 was not supported.

**FIGURE 3:**
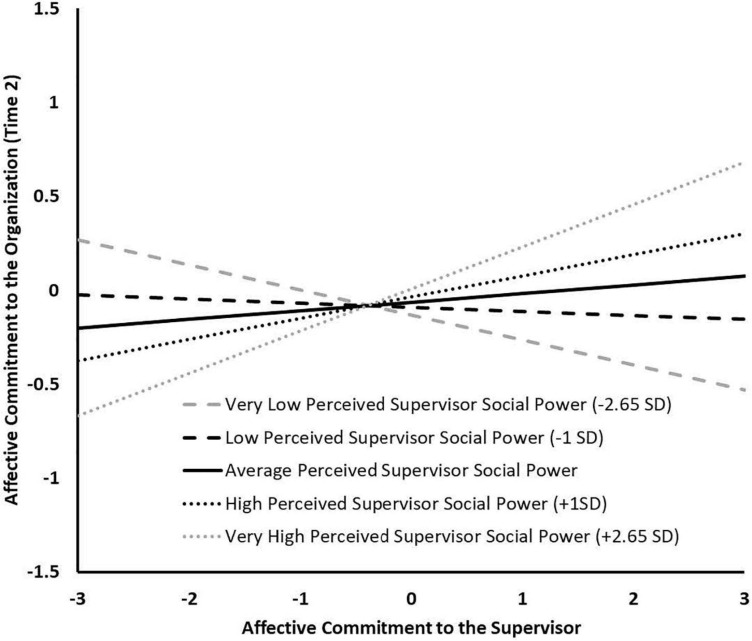
Study 3 Simple Slopes for the Moderation Effect of Perceived Supervisor Social Power between Affective Commitment to the Supervisor and Organizational Commitment.

As can be seen in Table 5 (bottom of the table), PSSP interacted with affective commitment to the supervisor to predict organizational commitment at Time 2 (β = 0.088, *p* < 0.05). The simple slopes analysis for this interaction indicated that the effect of affective commitment to the supervisor was significantly positive only at very high levels of PSSP (+2.65*SD*: *b* = 0.225, *SE* = 0.114, *p* < 0.05) but non-significant at lower levels of PSSP (+1*SD*: *b* = 0.113, *SE* = 0.087, *ns*; *M*: *b* = 0.046, *SE* = 0.078, *ns*; −1*SD*: *b* = −0.022, *SE* = 0.080, *ns*), as shown in Figure 3. Hypothesis 5 is further supported. In contrast, PSSP did not moderate the indirect relation between affective commitment to the supervisor and turnover intention through organizational commitment. Indeed, this indirect effect was non-significant even at very high levels of PSSP (indirect effect = −0.059, *SE* = 0.036, 95% bootstrap CI = −0.159, 0.001). Thus, Hypothesis 6 is rejected.

## Discussion

It has been theoretically posited that employees’ perceptions of their supervisor power integrate both influence attempts directed at themselves and the observation of influence attempts directed at others, and that both types of social information entail a potential to influence (Fiol et al., 2001; Farmer and Aguinis, 2005). However, research has disproportionally focused on the former, specifically investigating influence tactics used by supervisors to influence followers’ beliefs, attitudes, and behaviors (Yukl and Falbe, 1991; Yukl and Tracey, 1992; Carson et al., 1993; Yukl et al., 1993; Elangovan and Xie, 1999; Koslowsky et al., 2001; Pierro et al., 2013; Chi and Ho, 2014; Chiu et al., 2017; Chi et al., 2018; Haller et al., 2018; Peyton et al., 2019). This lack of research on the latter might in part be explained by the lack of a validated measure to assess observations of a supervisor’s power outside the supervisor-follower dyad.

This study contributed to fill this gap in the literature by proposing a new measure of PSSP that specifically focused on perceptions of supervisors’ power in the organization. A definition of PSSP was established and a five-item measure was developed. Results from three studies supported the factor validity and the psychometric soundness of this new scale. Specifically, PSSP unidimensional measurement model and reliability were supported in four samples of workers. Importantly, these results were cross-validated as PSSP has been shown to be invariant across three countries that differ in languages and on Hofstede’s (1991) power distance dimension, namely Canada (low power distance), France (intermediate power distance), and Romania (high power distance).

In addition, in *Study 2* we provided evidence for PSSP convergent and discriminant validity in relation to French and Raven’s (1959) power bases, supporting the idea that PSSP is related to, yet distinct, from followers’ perceptions of influence tactics directed at them, thus supporting Hypothesis 1. Similarly, the results from *Study 3* supported the idea that PSSP was related, yet distinct from, constructs characterizing the bond between the supervisor and the organization (i.e., perceptions of supervisor organizational support, and of supervisor-organization value congruence). PSSP was also shown to be related, yet distinct, from supervisors’ networking ability, an indicator of a supervisor’s capacity to develop influence throughout the organization. These results thus supported Hypothesis 2.

Finally, as recommended by MacKenzie et al. (2011), the nomological validity of the newly developed PSSP construct was verified by integrating it into a theoretical model involving affective commitment toward the organization, turnover intention, and commitment to the supervisor. In *Study 1* and *Study 2*, PSSP was positively related to employees’ organizational commitment, independently of the effect of affective commitment to the supervisor (i.e., a well-known predictor of organizational commitment; Vandenberghe and Bentein, 2009). However, Study 3 unexpectedly revealed a non-significant association between PSSP and organizational commitment, which is logically extended to the indirect relation between PSSP and turnover intention as mediated by organizational commitment. Thus, Study 3 results, respectively, offers a partial support to Hypothesis 3 and rejects Hypothesis 4. Incidentally, a direct, negative relation between PSSP and turnover intention was also observed in Study 3.

Furthermore, in all three studies, PSSP positively moderated the relation between affective commitment to the supervisor and organizational commitment, thus supporting Hypothesis 5. However, Study 3 showed that PSSP did not moderate the indirect relation between affective commitment to the supervisor and turnover intention through increased organizational commitment, thus rejecting Hypothesis 6. The present findings lead to a number of implications.

### Theoretical Implications and Contributions

Perceived supervisor social power offers new perspectives on how supervisor power can be understood. First, PSSP captures a unique construct domain, distinguishable from French and Raven’s (1959) power bases. When viewed through the lens of power bases, the application of supervisor social power is linked to the employee-supervisor dyad and addresses the means used by a supervisor to obtain compliance from subordinates to achieve work-related goals (Yukl et al., 1993; Koslowsky et al., 2001). PSSP does not involve specific means that supervisors might use to obtain compliance from subordinates. Rather, it refers in a broad sense to how employees perceive that their supervisor can influence decisions and important actors in the organization at large. However, it is interesting to note that *Study 2* data revealed moderate, positive correlations between PSSP and reward, legitimate, expert, and referent power but a negative correlation between PSSP and coercive power (Table 4). This may denote that the ability to use punishments or constraints (i.e., coercive power) to obtain subordinate compliance interferes with the perception of one’s supervisor having power in the larger organization. Furthermore, it could be possible that the higher PSSP, the less a supervisor needs to rely on coercive power and the more s/he will rely on more prosocial forms of power such as expert power. As such, prosocial bases of power could be more effective in influencing employees in the presence of high PSSP, making coercive power a last resort means of influence.

Second, studies have shown that supervisors’ centrality in the organization (Sparrowe and Liden, 2005; Venkataramani et al., 2010), supervisors perceived organizational identity (Eisenberger et al., 2002), and expressions of support by organizations towards supervisors (Shanock and Eisenberger, 2006) all influence employees’ attitudes. We reasoned that this research stream spoke to how supervisors’ ability to represent the organization is perceived by employees and that such perceptions mattered for employees’ sense of organizational identification and belongingness. Perceiving that their supervisors exert an influence on important organizational actors such as C-Suite members or owner(s) and on the organizational decision process (i.e., PSSP) is likely to lead employees to internalize the organizational goals and values and further see themselves as powerful representatives of the organization. In addition, through their supervisor’s strong status and connections to the organizational network, they might also think that they have access to a wider range of opportunities (Seibert et al., 2001; Venkataramani et al., 2010), which should further strengthen the employee-organization bond.

Third, consistent with the proposed influence mechanism, PSSP was found to be positively related to organizational commitment, controlling for affective commitment to the supervisor (a major predictor of organizational commitment) (*Study 1* and *Study 2*) and supervisor power bases (*Study 2*). This finding did not hold in *Study 3*, which controlled for related constructs (i.e., supervisor networking ability, supervisor perceived organizational support, and supervisor-organization value congruence) and sought to predict organizational commitment measured at a later point in time. Supervisor perceived organizational support and supervisor-organization value congruence were the two predictors of organizational commitment, while PSSP and affective commitment to the supervisor were unrelated to organizational commitment. This is interesting because it suggests that organizational commitment may be primarily driven by signs of support from the organization to supervisors and concrete indications of closeness between the supervisor and the organization. Thus, actions from, and defining features of, the organization appear to be central elements that contribute to organizational commitment. Of utmost importance, PSSP and affective commitment to the supervisor were both negative predictors of turnover intention, controlling for supervisor networking ability, supervisor perceived organizational support, and supervisor-organization value congruence, indicating that by themselves these variables prevent employees from engaging in withdrawal tendencies. It is conceivable that in the context of a high-power distance country (i.e., Romania; Hofstede, 1991; see text footnote 1), the mere possibility that an employee might internalize his/her supervisors’ power into his/her identity (Fiol et al., 2001), thus feeling more powerful, might in itself be gratifying enough for employees to reduce their withdrawal cognitions. However, future research should further examine the actual role of power distance in the relation between PSSP, commitment, and turnover intention. Future research is also needed to examine if these effects extend to actual turnover.

Finally, PSSP enhanced the relation between affective commitment to the supervisor and organizational commitment. This finding is in line with other research that found supervisor-organization value congruence to moderate the relation between affective commitment to the supervisor and organizational commitment, and indirectly turnover (Vandenberghe et al., 2017). Perceiving one’s supervisor to hold power in the organization helps transfer affective commitment to the supervisor into organizational commitment. Supervisors are expected to endorse roles that are central in the structural functioning of the organization and transcend their individuality (Sluss and Ashforth, 2007). When they are perceived as being influential and at the center of the decisional process (i.e., high PSSP), followers might perceive that their supervisor more fully endorses those expected roles and thus more fully embodies the organization’s goals and values. This possible effect might facilitate the transfer of affective commitment from the supervisor to the organization.

### Exploration of PSSP Boundary Conditions

As this paper presented a conceptual framework for PSSP and offered a psychometrically sounded scale to measure this construct, we believe it is critical for future studies to investigate the boundary conditions of its influence on followers. More precisely, its interactive effect with supervisors’ behaviors directed toward followers, sex, and cultural characteristics should be explored.

First, PSSP resides outside of the supervisor-follower relationship. Therefore, it is independent from actual attempts to influence the target employee through leadership behaviors. However, PSSP could also moderate supervisors’ attempts to influence the target employees by enhancing the positive or negative effects of these attempts. According to Abele and Wojciszke (2007), in an interdependent dyadic relationship, dependent individuals pay close attention to the agentic characteristics of the other person upon which the achievement of their goals and well-being depends. They do so because such attributes could be either profitable or harmful for them depending on the intentions, goals, and behaviors of the other person in the relationship. In other words, PSSP coupled with constructive and prosocial leadership behaviors directed toward followers such as transformational or empowering leadership might send the message that the supervisor will use his or her power in the organization to facilitate the achievement of their objectives. On the contrary, PSSP coupled with destructive behaviors such as petty tyranny or abusive supervision might send the message that an influent member of the organization is hindering followers’ goal achievement. Future studies should explore how PSSP and supervisory behaviors directed toward employees interact in predicting employee outcomes.

Second, research should further study the moderating role of gender (as well as that of other possible individual moderators) in the relationship between PSSP and employee outcomes. Attributes characterized as agentic, such as power, are generally associated with the stereotype of masculinity in supervisory positions (Johnson et al., 2008; Koenig et al., 2011). Furthermore, it has been shown that women occupying supervisory roles who act in a counter-stereotypical fashion tended to be unjustifiably depreciated by their followers (Eagly et al., 1992; Eagly and Karau, 2002). For this reason, it remains possible for women supervisors perceived has having a high level of power (i.e., high PSSP) to be perceived in a less favorable manner than their male counterparts by their subordinates, which might in turn hinder the benefits of PSSP. Similarly, research has shown that followers’ characteristics, such as gender or personality traits, may influence how they perceive their supervisor’s behaviors (Wang et al., 2019).

Finally, research on PSSP should further explore how PSSP and its influence on employees generalize or differ across cultures. As previously stated, our results supported the measurement invariance of responses to the PSSP questionnaire across the cultures considered in the present study, which are known to differ in terms of power distance. However, the relations between PSSP and employee attitudes and intentions were found to be less clear in a culture characterized by a high-power distance (i.e., Romania). Furthermore, PSSP, as well as its relations with other constructs, might differ depending on whether it is assessed in the context of an individualistic vs. collectivistic culture. Indeed, Torelli and Shavitt (2010) showed that power is perceived as something to be used for advancing a person’s own objectives and status in more individualistic cultures. On the contrary, in collectivistic cultures, power is perceived as something used to benefit others. Thus, PSSP could have a more positive influence in more collectivistic countries. Future studies should further explore the influence of PSSP across cultures, particularly among non-Western cultures given that the focus of the present article was limited to participants from North American and European cultures.

### Strengths and Limitations

The present set of studies provides evidence for the validity and measurement invariance of a new measure of PSSP across four samples from three countries in three languages. The convergent and discriminant validity of this new measure was also supported in relation to multiple related variables. However, limitations should be considered. First, we did not measure objective employee turnover. Thus, it is unclear whether the effect of PSSP on turnover intention (Study 3) would extend to turnover. Second, the PSSP scale has been developed from employee perceptions of supervisor social power. Future research might consider, through a multilevel analytical perspective, whether such perceptions are shared across employees from the same teams and could define a climate for supervisor social power. Such climate may influence how employee attitudes within their teams generalize to organizational commitment. Similarly, it would be interesting to explore whether PSSP actually predicts how decisions are taken in the organization. Likewise, other beneficial outcomes of affective commitment should be investigated, such as performance and citizenship behaviors (Meyer et al., 2002). It would also be important to assess the role of power distance in the relation between PSSP and affective organizational commitment.

## Data Availability Statement

The raw data supporting the conclusions of this article is available upon request from the authors.

## Ethics Statement

The studies involving human participants were reviewed and approved by the Comité d’éthique de la recherche, HEC Montréal. The participants provided their written informed consent to participate in this study.

## Author Contributions

LC-P and CV wrote the manuscript’s theoretical introduction and discussion. LC-P and AM performed the statistical analyses and wrote the related sections. CV contributed to the acquisition of data. All authors contributed to the revision and the improvement of the manuscript. All authors listed above contributed to the design and the interpretation of the results.

## Conflict of Interest

The authors declare that the research was conducted in the absence of any commercial or financial relationships that could be construed as a potential conflict of interest.

## Footnotes

1 https://www.hofstede-insights.com/product/compare-countries/

2 In the present studies, a variable reflecting participants’ hierarchical status (coded 0 for employee and 1 for manager) was available in three of the data sets (Canada- French, Canada- English, Romania). When this variable was added as a control to the prediction, the results remained virtually unchanged, and the conclusions identical to those reported in the present study (these additional results are available upon request from the authors).
